# Targeted knockdown of ATM, ATR, and PDEδ increases Gag HIV-1 VLP production in HEK293 cells

**DOI:** 10.1007/s00253-024-13389-8

**Published:** 2025-01-02

**Authors:** Andy Díaz-Maneh, Pol Pérez-Rubio, Cristina Rigau Granes, Laia Bosch-Molist, Jesús Lavado-García, Francesc Gòdia, Laura Cervera

**Affiliations:** 1 Grup d’Enginyeria de Bioprocessos i Biocatàlisi Aplicada, ENG4BIO, Escola d’Enginyeria, Universitat Autònoma de Barcelona, Campus de Bellaterra, Cerdanyola del Vallès, 08193 Barcelona, Spain; 2 Aglaris Cell, C/ Santiago Grisolía, 2, Tres Cantos, 28760 Madrid, Spain; 3 Asklepios Biopharmaceutical, Inc, 20 TW Alexander Dr #110, Research Triangle Park, Chapel Hill, NC 27709 USA; 4 Novo Nordisk Foundation Center for Biosustainability, Technical University of Denmark, 2800 Kgs. Lyngby, Denmark; 5 Serra Hunter, Catalonia, Barcelona, Spain

**Keywords:** VLP, HEK293, Transfection, ShRNA, ATM

## Abstract

**Abstract:**

Several strategies have been developed in recent years to improve virus-like particle (VLP)-based vaccine production processes. Among these, the metabolic engineering of cell lines has been one of the most promising approaches. Based on previous work and a proteomic analysis of HEK293 cells producing Human Immunodeficiency Virus-1 (HIV-1) Gag VLPs under transient transfection, four proteins susceptible of enhancing VLP production were identified: ataxia telangiectasia mutated (ATM), ataxia telangiectasia and rad3-related (ATR), DNA-dependent protein kinase catalytic subunit (DNA-PKcs), and retinal rod rhodopsin-sensitive cGMP 3',5'-cyclic phosphodiesterase subunit delta (PDEδ). The knockdown of ATM, ATR, and PDEδ in HEK293 cells increased HIV-1 VLP titers in the supernatant by 3.4-, 2.1-, and 2.2-fold, respectively. Also, possible metabolic synergies between plasmids were investigated by statistical design of experiments (DoE), enabling us to identify the optimal production strategy, that was further demonstrated at lab-scale stirred tank bioreactor operated in perfusion, significantly increasing both VLPs specific and volumetric productivities to 8.3 × 10^3^ VLPs/cellxday and 7.5 × 10^12^ VLPs/Lxday, respectively.

**Key points:**

*• ATM, ATR, and PDEδ knockdowns increased VLP production in HEK293 cells.*

*• Knockdown of ATM increased budding efficiency and extracellular vesicle concentration.*

*• ATM knockdown could be intensified to bioreactor scale operated in perfusion.*

**Graphical Abstract:**

**Figure:**
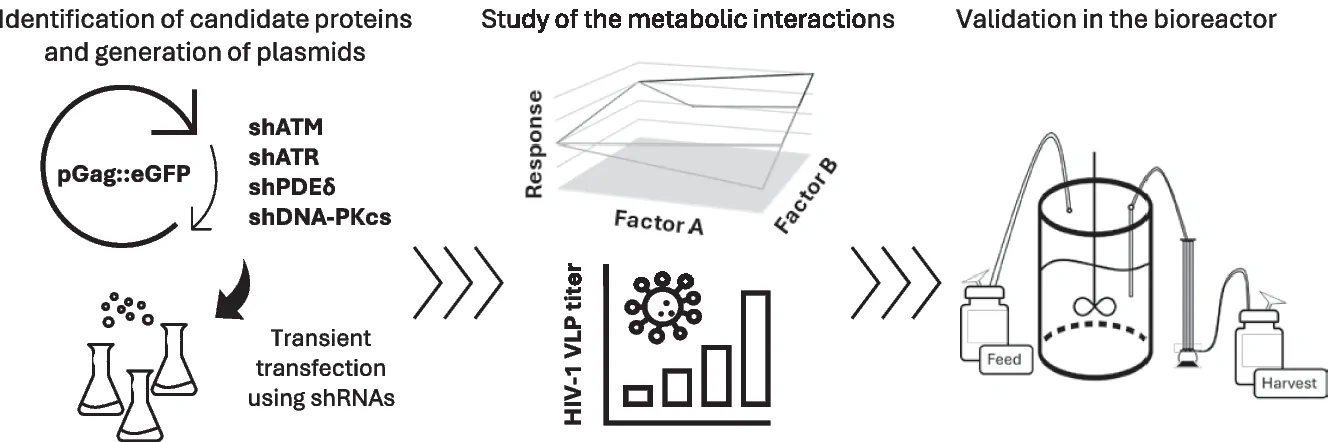

## Introduction

The demand for developing versatile, safe, and efficient platforms for vaccine production is rising in the scientific community and society. Pandemics such as SARS-CoV-2, as well as seasonal influenza virus outbreaks (e.g., Zika and Ebola), have shown the importance of the improvement of vaccine production processes to swiftly respond to these challenges.

Virus-like particles (VLPs) have been gaining ground in recent years as safe and efficient candidates for antigen presentation. The Gag polyprotein of Human Immunodeficiency Virus-1 (HIV-1) is capable of self-assembling and being secreted from host cells through a process known as budding, giving rise to particles morphologically similar to the immature virions of HIV-1. These particles do not contain genetic material, thus being non-infective and safe (Roldão et al. 2010; Crisci et al. 2012).

As HIV, Gag VLPs are embedded in a lipidic envelope. Due to this characteristic, animal cells are often selected as a platform for their recombinant production. HEK293 cells show low expression of the interferon-induced gene *BST2* and are therefore considered a permissive cell line. In addition, they are able to grow in suspension and under serum-free conditions, can be easily transfected, and provide the required post-translational machinery for the proper expression, transport, assembly, and budding of HIV-1 VLPs (Perez-Caballero et al. 2009; Tan et al. 2021). For this reason, HEK293 cells have been extensively used in the production of Gag VLPs (Fuenmayor et al. 2017).

Two major strategies for producing recombinant proteins in mammalian cells have been described so far: stable expression and transient transfection. In transient transfection, a vector that encodes for a certain protein is introduced into the cells using transfecting agents such as polyethyleneimine (PEI) or lipofectamine. This is the preferred approach in the initial phases of research and early development, in which screening of several therapeutic candidates is needed. It is also currently employed in the biomanufacturing of complex products such as viral vectors for gene therapy (Tan et al. 2021). In this work, PEI-mediated transient transfection of HEK293 cells will be used to produce VLPs.

Among the aspects of the upstream process that can be optimized to improve production yields, the optimization of the operational strategy and the cellular metabolic state emerge as some of the most important. Regarding the cultivation mode, perfusion, consisting of constantly feeding fresh media while removing spent media and by-products has proven to be highly efficient for cell-based bioprocesses intensification (Bielser et al. 2018). The so-called extended gene expression (EGE) protocol is an approach that enables to partially overcome plasmid loss and to maintain higher specific production rates by means of medium replacements and cell retransfections. This alternative perfusion approach has already been implemented at bioreactor scale for Adeno-Associated Virus (AAV) and VLP production (Cervera et al. 2015b; Lavado-García et al. 2020a; Godia-Casablancas et al. 2022).

From the cellular metabolic state perspective, it is highly relevant to determine which are the metabolic bottlenecks hindering production, as a previous step to the development of strategies to increase product titers. Concerning the production of HIV-1 VLPs in HEK293 cells, there are two major constraints: the production of large amounts of the Gag polyprotein and the cellular trafficking and assembly of this protein, which leads to VLP formation and secretion. These limitations have been addressed by researchers using production enhancers to increase the production of HIV-1 VLPs in HEK293 cells (Cervera et al. 2015a). Nonetheless, toxicity, economic cost, regulatory constraints, and the need to eliminate these additives in the downstream process have led to the exploration of alternatives to their use. In this context, the shRNA-mediated knockdown to mimic the effect of additives has been demonstrated (Fuenmayor et al. 2018).

Caffeine is among those additives described to increase Gag VLP titers (Ellis et al. 2011). This naturally occurring methylxanthine is an inhibitor of several proteins, some of them canonical phosphodiesterases (PDEs), ataxia telangiectasia mutated (ATM), ataxia telangiectasia and rad3-related (ATR), or the DNA-dependent protein kinase catalytic subunit (DNA-PKcs) (Choi et al. 1988; Sarkaria et al. 1999; Block et al. 2004). All of them have been identified in the proteome of HEK293 cells under transient transfection producing Gag VLPs (Lavado-García et al. 2020b). Also, ATM, ATR, and DNA-PKcs have been described to be related somehow to the replication of HIV-1 in mammalian cells (Roshal et al. 2003; Nunnari et al. 2005; Ellis et al. 2011).

In this work, the effect of the knockdown of ATM, ATR, PDEδ and DNA-PKcs proteins on HIV-1 VLPs production is explored. The best candidate is then employed together with the optimized EGE protocol described earlier to obtain an intensified upstream VLP production process.

## Materials and methods

### HEK293 mammalian cell line and culture conditions

The cell line used in this work is a serum-free suspension-adapted HEK293 cell line (HEK293SF-3F6) kindly provided by Dr. Amine Kamen from the National Research Council of Canada (NRC, Montreal, Canada). Cells were cultured in non-baffled disposable polycarbonate 125 mL flasks with a vent cap (Corning, Somerville, MA, USA) at 37 °C, 5% of CO_2_, 20 mL of working volume, and 85% relative humidity at 130 rpm in an LT-X Kuhner shaker (LT-X Kuhner, Birsfelden, Switzerland). The culture media was HyClone HyCell TransFx-H Transfection Medium (GE Healthcare, Life Sciences, Chicago, IL, USA) supplemented with 4 mM GlutaMAX and 0.1% Pluronic F-68 non-ionic surfactant (Gibco, Life Technologies, Thermo Fisher, San Jose, CA, USA).

Cell concentration and viability were assessed using the NucleoCounter NC-3000 automatic cell counter (Chemometec, Allerod, Denmark) according to the manufacturer’s instructions.

### Transient transfection

HEK293 suspension cells were transiently transfected using PEIpro (Polyplus-transfection, Illkirch-Graffenstaden, France). Transfections were carried out at a cell density of 2 × 10^6^ cells/mL and a final DNA concentration of 1 μg/mL. PEI/DNA complexes were formed by adding PEI to plasmid DNA diluted in fresh culture media (10% of the total culture volume to be transfected). Briefly, the pGag::eGFP plasmid was diluted with supplemented HyCell media and vortexed for 10 s. PEI was added in 1:2 (w/w) DNA/PEI ratio and vortexed three times, then the mixture was incubated for 15 min at room temperature and added to the cell culture. All plasmids used in the transfection experiments were amplified using *Escherichia coli* (DH5α strain, Thermo Fisher, San Jose, CA, USA) and purified using the QIAGEN Plasmid *Plus* Mega Kit (Qiagen Inc., Hilden, Germany) (Mercedes Segura et al. 2007).

Extended gene expression was carried out as previously described (Lavado-García et al. 2020c). Briefly, cells were transfected as described above and retransfected at 24 hpt with 1.7 μg/mL of DNA (DNA:PEI of 1:2). Medium replacement was performed following a semi-perfusion strategy at a constant cell-specific perfusion rate (CSPR) of 30–60 pL/cell × day before retransfection and then every 24 h, depending on viable cell density.

### Design of experiments: Box-Behnken

A 27-experiments Box-Behnken design with 3 central points was performed. The experimental matrix along with the response of each of the runs are shown in Table 1. DNA:PEI ratio was always maintained at 1:2. ANOVA analysis and surface plots of the model, as well as Fit statistics, were calculated using Design-Expert 11 (Stat-Ease Inc., Minneapolis, MN, USA). Briefly, Anova analysis indicates the significance for each term of the equation, the Adjusted *R*^2^ indicates the proportion of variance in the response explained by the factors adjusted by the number of factors, and the lack of fit *p*- and *F*-values indicate that the experimental factors adequately predict the response. The model equation containing the constant, as well as the single terms and interactions affecting the response, can also be found in Table 1.

**Table 1:** Box-Behnken matrix of coefficients, results, ANOVA analysis and fit statistics for the optimization of the plasmid ratios to maximize VLP concentration in the supernatant

Run	DNA concentration (µg/mL)				Response
	ATM	ATR	PDE	HDAC	Relative Fluorescence Units (RFU)
1	0.5	0.3	0.1	0.3	165
2	0.3	0.5	0.5	0.3	134
3	0.5	0.1	0.3	0.3	171
4	0.3	0.1	0.3	0.1	148
5	0.5	0.5	0.3	0.3	133
6	0.3	0.5	0.3	0.1	162
7	0.5	0.3	0.3	0.1	172
8	0.3	0.5	0.1	0.3	167
9	0.1	0.1	0.3	0.3	127
10	0.3	0.1	0.1	0.3	155
11	0.3	0.3	0.3	0.3	148
12	0.5	0.3	0.3	0.5	131
13	0.3	0.3	0.3	0.3	162
14	0.5	0.3	0.5	0.3	136
15	0.1	0.3	0.5	0.3	143
16	0.1	0.3	0.3	0.5	145
17	0.1	0.3	0.3	0.1	137
18	0.3	0.3	0.3	0.3	127
19	0.3	0.3	0.3	0.3	156
20	0.3	0.3	0.1	0.5	160
21	0.3	0.3	0.5	0.1	156
22	0.3	0.3	0.5	0.5	106
23	0.1	0.5	0.3	0.3	142
24	0.3	0.1	0.5	0.3	153
25	0.3	0.1	0.3	0.5	156
26	0.3	0.3	0.1	0.1	155
27	0.3	0.5	0.3	0.5	133
28	0.1	0.3	0.1	0.3	150
29	0.3	0.3	0.3	0.3	156
Code		-1	0	1	
[DNA] (mg/mL)		0.1	0.3	0.5	

Analysis of Variance and Equation			
	***F***-value	***p***-value	Equation
**Model**	5.47	0.0027	RFU
Constant			45.7
ATM	4.76	0.0497	261.2
ATR	1.69	0.2182	162.2
PDE	18.06	0.0011	100.3
HDAC	11.72	0.005	213.3
ATM·ATR	10.1	0.0079	-334.1
ATM·PDE	1.56	0.236	-131.1
ATM·HDAC	8.5	0.013	-306.4
ATR·PDE	3.29	0.0947	-190.7
ATR·HDAC	4.93	0.0465	-233.3
PDE·HDAC	10.53	0.007	-341.1
ATM2	0.0035	0.9541	-5.4
ATR2	0.8182	0.3835	82.3
PDE2	0.7411	0.4062	78.4
HDAC2	0.0295	0.8665	15.6
**Lack of Fit**	0.187	0.9737	
**Fit statistics**			
	***R***^**2**^	0.8646	
	**Adjusted *R***^**2**^	0.7067	

### Flow cytometry

Samples were taken every 24 h, and cells were fixed with 2% formaldehyde for 10 min, centrifuged, and then resuspended in phosphate-buffered saline for fluorescence-activated cell sorting (FACS) analysis. The percentage of green fluorescence protein (GFP)-positive cells was assessed using a BD FACS Canto flow cytometer (BD Biosciences, San Jose, CA, USA). 488 nm laser was used for GFP measurement. The results were analyzed with the FACS DIVA software (BD Biosciences, San Jose, CA, USA).

### Plasmids and shRNAs

The pGag::eGFP plasmid codes for HIV-1 Gag polyprotein fused in frame with the enhanced green fluorescence protein (eGFP), allowing easy quantification of VLPs (Hermida-Matsumoto and Resh 2000). This is a translational gene fusion of the HIV *gag* gene and *egfp* gene under a Cytomegalovirus (CMV) enhancer and a CMV promoter with no IRES sequence between them. The plasmid pGag::eGFP-shHDAC5 was used as a backbone to generate the plasmids for the shRNAs, that were inserted downstream of the U6 promoter, replacing the shRNA against HDAC5 (Fuenmayor et al. 2018).

All oligos and ultramers used were obtained from Integrated DNA Technologies (Coralville, IA, USA) and are listed in Table 2. All plasmids were verified using PCR, followed by agarose gel electrophoresis, and Sanger sequencing in the Institut de Biotecnologia I Biomedicina (Universitat Autònoma de Barcelona, Catalonia, Spain) sequencing service using oligos 10 and 11. All transformations were done in chemically competent DH5⍺ *E. coli*., that were selected in plates using Kanamycin antibiotic (50 µg/mL). DNA was purified with Invitrogen™ PureLink™ Quick Plasmid Miniprep Kit (Fisher Scientific, Hampton, NH, USA).

**Table 2:** Sequence of primers, ultramers, and shRNAs used for the generation of the plasmids

Code	Name	Classification	Sequence (5’−3’)
1	**pGag::eGFP-shHDAC5 F Gibson Assembly**	Amplification primer	ATGGAGTTCCGCGTTACATA
2	**pGag::eGFP-shHDAC5 R Gibson Assembly**	Amplification primer	GGTGTTTCGTCCTTTCCACAAGATA
3	**pcDNA3.1( +)-shATM F Gibson Assembly**	Amplification primer	GTGGAAAGGACGAAACACCGGGAGCGATTGTAGCAACAT
4	**pcDNA3.1( +)-shATM R Gibson Assembly**	Amplification primer	ATGTAACGCGGAACTCCATGAGACCCAAGCTGGCTAGCGTT
5	**pcDNA3.1( +)-shATR F Gibson Assembly**	Amplification primer	GTGGAAAGGACGAAACACCAACCTCCGTGATGTTGCTTG
6	**pcDNA3.1( +)-shATR R Gibson Assembly**	Amplification primer	ATGTAACGCGGAACTCCATGAGACCCAAGCTGGCTAGCGTT
7	**pcDNA3.1( +)-shDNA-PKcs F Gibson Assembly**	Amplification primer	GTGGAAAGGACGAAACACCCTTTATGGTGGCCATGGAGGTGAA
8	**pcDNA3.1( +)-shDNA-PKcs R Gibson Assembly**	Amplification primer	ATGTAACGCGGAACTCCATGAGACCCAAGCTGGCTAGCGTT
9	**Ultramer shRNA-PDEδ**	Ultramer sDNA	GTGGAAAGGACGAAACACCGCACATCCAGAGTGAGACTTTCTCGAGAAAGTCTCACTCTGGATGTGCTTTTTGATGGAGTTCCGCGTTACATA
10	**pGag::eGFP-shRNA F seq**	Sequencing primer	TTGTGGAAAGGACGAAACAC
11	**pGag::eGFP-shRNA R seq**	Sequencing primer	TGGAAAGTCCCTATTGGCGTTA
12	**shATM**	Short-hairping RNA sequence	GGGAGCGATTGTAGCAACATAAGGTGAAGCCACTTATGTTGCTACAATCGCTCCCTTTTTG
13	**shATR**	Short-hairping RNA sequence	AACCTCCGTGATGTTGCTTGAGTGAAGCCATCAAGCAACATCACGGAGGTTTTTTTG
14	**shPDE**	Short-hairping RNA sequence	GCACATCCAGAGTGAGACTTTCTCGAGAAAGTCTCACTCTGGATGTGCTTTTTG
15	**shDNA**	Short-hairping RNA sequence	CTTTATGGTGGCCATGGAggtgaaGCCACTCCATGGCCACCATAAAGTTTTTG
16	**shScramble 1**	Short-hairping RNA sequence	CCTAAGGTTAAGTCGCCCTCGGTGAAGCCACGAGGGCGACTTAACCTTAGGTTTTT
17	**shScramble 2**	Short-hairping RNA sequence	CCGCAGGTATGCACGCGTGTGAAGCCAACGCGTGCATACCTGCGGTTTTT
18	**shScramble 3**	Short-hairping RNA sequence	GGAAGAGCGAGCTCTTCTGTGAAGCCAAGAAGAGCTCGCTCTTCCTTTTT

ATM, ATR, and DNA-PKcs shRNAs were obtained and cloned into the pcDNA3.1( +) plasmid (GenScript Biotech Corporation, NJ, USA). They were amplified by PCR (using oligos 3–8) downstream of the U6 promoter of the pGag::eGFP-shHDAC5 plasmid, amplified with oligos 1 and 2. The PCR-amplified fragments were run on an agarose gel and purified with the QIAquick Gel Extraction Kit (Qiagen Inc., Hilden, Germany). DNA concentration and quality of purified bands were measured with Nanodrop 1000 (Thermo Fisher Scientific, Waltham, MA, USA). Fragments were assembled following a Gibson Assembly protocol: 1-h incubation at 50 °C using the Hi-Fi Master Mix 2x (New England Biolabs, Ipswich, MA, USA) (Gibson et al. 2009). The molar ratio of backbone:insert used was 1:5 for all 3 cases and was calculated using Eq. 1:

1 $$pmols DNA=\frac{\left(ng of DNA\right)x1000}{base pairsx650 Da}$$

The shRNA from PDEδ has been described in the literature (Zimmermann et al. 2013) and was obtained as ssDNA ultramer flanked by regions with sequence complementarity to the adjacent regions where it was going to be inserted in the pGag::eGFP-shHDAC5-based backbone (Table 2). It was cloned downstream from the U6 promoter following a protocol like the one explained above, slightly adapted to assemble ssDNA (Ma et al. 2014; Jacobs et al. 2015). Three different shRNA scrambles cloned into the pGag::eGFP backbone were obtained from GenScript.

### HIV-1 gag VLP quantification

#### Fluorimetry

The concentration of HIV-1 Gag VLPs was assessed by fluorimetry using an in-house developed and validated quantification assay (Gutiérrez-Granados et al. 2013). VLP-containing supernatants were recovered by cell culture centrifugation at 1000 g for 5 min. Relative fluorescence unit (RFU) values were calculated by subtracting the fluorescence unit values of non-transfected negative control samples. There is a linear correlation between fluorescence intensity and p24 values determined using Innotest ELISA HIV antigen mAb (Innogenetics NV, Gent, Belgium). RFU values can be converted to Gag::eGFP concentration values, using Eq. 2, and in VLPs/mL, using Eq. 3:

2 $$Gag\colon\colon eGFP \left(\frac{ng}{mL}\right)=\left(3.245xR.F.U-1.6833\right)x36$$

3 $$\frac{VLPs}{mL}=\left(4.448xR.F.U-63.3\right)x{10}^{8}$$

#### Nanoparticle tracking analysis

Nanoparticle tracking analysis (NTA) was employed for VLP characterization using a NanoSight®NS300 (Nanosight Ltd, Amesbury, UK) at the Preparation and Characterization of Soft Material services at the Institut de Ciència de Materials de Barcelona (ICMAB-CSIC, Bellaterra, Spain). Sample analyses were performed as previously described (Gutiérrez-Granados et al. 2013).

#### Flow virometry

HIV-1 Gag::eGFP VLPs were quantified using a CytoFLEX LX (Beckman Coulter, Brea, CA, USA). VLPs were detected based on violet side scatter (V-SSC) and Fluorescein IsoTioCyanate (FITC) fluorescence signals. Laser gains were set to 72 for forward scatter (FSC), 135 for side scatter (SSC), 9 for V-SSC, and 500 for FITC. Prior to analyses, samples were diluted in filtered PBS to achieve a concentration of 500–5,000 events/µL ensuring an abort rate below 5%. A minimum of 20,000 VLP events were recorded at a flow rate of 10 µL/min. VLPs were distinguished from background noise using V-SSC vs B525-FITC density plots. Results were normalized employing an internal control. Data analysis was conducted using CytExpert v.2.3 software (Beckman Coulter, Brea, CA, USA).

### Statistical analyses

Statistical analyses were performed using the statistical analysis tool of GraphPad Prism 8 software (GraphPad Software Inc., San Diego, CA, USA).

### Western blots

Twenty microliters of each condition was prepared and separated on an SDS–polyacrylamide gel electrophoresis and transferred onto a polyvinylidene difluoride membrane for 7 min using the system Trans-Blot Turbo Transfer System (Bio-Rad, Hercules, CA, USA) as described in the manufacturer’s instructions. Membranes were incubated overnight with diluted primary antibody (described below) in 5% (*w/v*) skimmed dry milk 1 × Tris-buffered saline (PBS) 0.1% Tween-20 at 4 °C with gentle shaking. After primary incubation, a secondary incubation was performed using anti-rabbit IgG coupled with alkaline phosphatase antibody produced in goat (A9919, Burlington, Sigma-Aldrich, MA, USA) and anti-mouse IgG coupled with alkaline phosphatase antibody produced in goat (A3562, Sigma-Aldrich, Burlington, MA, USA), as required, in 2.5% (*w/v*) nonfat dry milk 1 × PBS 0.1% Tween-20 for 1 h at room temperature. Final membrane incubation with Clarity Western ECL substrates (Bio-Rad, Hercules, CA, USA) allowed the chemiluminescence protein band to be revealed when exposed in a ChemiDoc MP (Bio-Rad, Hercules, CA, USA). Images were analyzed using the software ImageJ (Schneider et al. 2012).

Primary antibodies used for protein validation were rabbit anti-ATR antibody (ab10312, Abcam, Cambridge, UK, 1:1000), rabbit anti-ATM antibody (ab32420, Abcam, Cambridge, UK), rabbit anti-DNA-PKcs antibody (ab32566, Abcam, Cambridge, UK, 1:1000), rabbit anti-PDEδ antibody (ab96825, Abcam, Cambridge, UK, 1:2500). Mouse anti-ß-actin antibody (MA5-15,739, Thermo Fisher Scientific, Waltham, MA, USA, 1:5000) was used as a loading control.

### Bioreactor culture conditions and setup description

A BioStat B Plus bioreactor (Sartorius Stedim Biotech, Goettingen, Germany) equipped with a 3-blade segment dual impeller with UP-DP configuration (Buffo et al. 2016) was used for HEK293 cell cultivation. The agitation was set at 200 rpm. The temperature was set at 37℃. pH was set at 7.1 and controlled with CO_2_ and NaHCO_3_ (7.5% *w/v*). Dissolved oxygen was controlled at 40% of air saturation by supplementing air with a sparger at a constant flow of 0.1 L/min and additional pure oxygen when needed. HEK293 growing exponentially in disposable polycarbonate 1-L shake flasks (Corning, Somerville, MA, USA) were used to seed the bioreactor at 0.5 × 10^6^ cells/mL in 1.5 L. Samples were taken every 24 h for cell counting and viability determination. After transfection, an assessment of the percentage of GFP-positive cells and VLP quantification was also performed every 24 h. Perfusion was achieved using an alternating tangential flow (ATF) cell retention device (Repligen, Waltham, MA, USA) with 0.2 µm pore size and 0.13 m^2^ of filtration area hollow fiber modules (Repligen, Waltham, MA, USA) and an ATF flow rate of 0.6 L/min. ATF filter was used to retain and concentrate VLPs inside the bioreactor vessel, allowing for cell retention and media replacement. When performing transfection, perfusion was stopped to incubate the cells with the DNA/PEI solution and reestablished 2 h after transfection (Cervera et al. 2017; Lavado-García et al. 2020a). To carry out media replacement, the filtration rate was set at 0.26 mL/min at the beginning of the process using a MasterFlex L/S peristaltic pump (MasterFlex Group, Gelsenkirchen, Germany) and was modified every day depending on the viable cell density to maintain a cell-specific perfusion rate (CSPR) of 30–60 pL/cell×day. The bioreactor and the ATF system were placed over a scale (MSE36200S-000-D0 Cubis, Sartorius, Goettingen, Germany) to control the mass displacement created due to the filtration of spent media. The scale was connected to a Scilog peristaltic pump (Scilog tandem 1081, Parker, Oxnard, CA, USA) which controlled the addition of fresh media upon detection of mass displacement to maintain constant volume in the bioreactor.

## Results

### *Knocking down ATM, ATR, and PDE*δ* increases VLP titers in the supernatant*

**Fig. 1:**
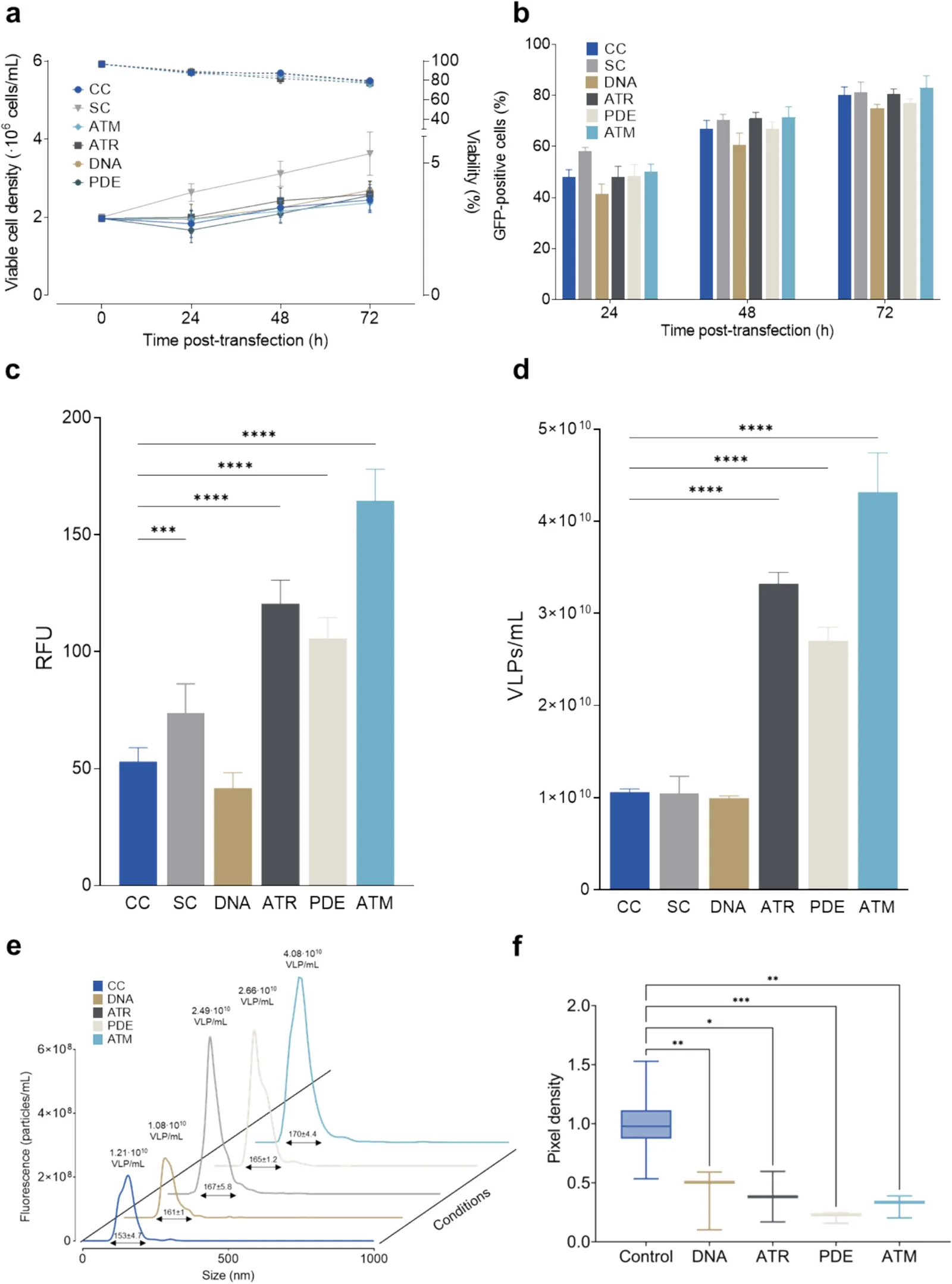
Growth kinetics and VLP production of HEK293 cells transfected with Gag::eGFP plasmid carrying the shRNAs targeting DNA-PKcs, ATR, ATM, and PDEδ. a. Viable cell density (solid lines) and viability (dotted lines) of the different conditions along the studied time course (*n* = 3). b. Transfection efficiency measured as percentage of GFP-positive cells by flow cytometry. c. VLP production of the different studied conditions measured via fluorometry as relative fluorescence units (RFU) in the supernatant at 72 hpt. d. VLP titers in the supernatant measured by flow virometry. e. Particle size distribution in the VLP-containing supernatants measured by nanoparticle-tracking analysis at 72 hpt (*n* = 3). VLP titers are indicated over the peaks. f. Pixel density analysis of western blot of the relative expression of ATM, ATR, DNA-PKcs and PDEδ proteins compared to CC and normalized using β-actin as housekeeping protein. In all panels: blue is for control transfected without any shRNA (CC), light grey for condition transfected with plasmid carrying a scramble shRNA (SC), brown for shRNA against DNA-PKcs (DNA), dark grey for shRNA against ATR (ATR), pearl for shRNA against PDEδ (PDE) and cyan for shRNA against ATM (ATM). Error bars indicate the standard deviation. Statistical significance studied using one-way ANOVA and Dunnett test analysis of the VLPs in the supernatant at 72 hpt of the knockdowns relative to the control condition (’****’ = *p*-value < 0.0001, ‘***’ = *p*-value = 0.0003)

The impact of ATM, ATR, PDEδ, and DNA-PKcs knockdown on the biosynthesis of Gag-based VLPs in HEK293 cells was investigated via transient transfection, as described above. One condition solely expressing the Gag polyprotein (CC) and another condition expressing the Gag polyprotein along with the U6 promoter and a scrambled shRNA sequence (SC) were used as controls. Cell density, viability, and transfection efficiency were monitored in each tested condition. Additionally, the concentration of VLPs present in the supernatant was quantified using fluorimetry and nanoparticle tracking analysis. Furthermore, the expression levels of the targeted proteins following knockdown were assessed via western blot analysis (Fig. 1f).

Initially, all conditions were transfected at 2 × 10^6^ cells/mL and their viable cell density exhibited similar trends, showing a slight increase to 2.5 × 10^6^ cells/mL by 72 h post-transfection (hpt) (Fig. 1a). Viability rates declined from > 90% at the onset of the culture to approximately 80% at 72 hpt in all conditions. The percentage of transfected cells (Fig. 1b) reached 80% across all conditions with no statistically significant differences observed between groups at the 72-h time point (Fig. 1b).

Measurement of VLP production was conducted both through assessment of RFU in the supernatant via fluorometry (Fig. 1c), as well as VLPs/mL quantification utilizing flow virometry (Fig. 1d). Notably, trends in VLP production were highly consistent between both methodologies. At 72 hpt, ATM, ATR, and PDE knockdown conditions yielded 165, 121, and 106 RFU, respectively, which were significantly higher (*p* < 0.0001) compared to the 53 RFU observed in the control condition. On the other hand, ATR, PDE, and ATM knockdown led to comparable increases in VLP titers in the supernatant, with respective values of 3.3, 2.7, and 4.3 × 10^10^ VLPs/mL (*p* < 0.0001). A much less significant effect was observed for DNA knockdown and SC conditions (42 and 74 RFU). These differences were not reflected in direct VLPs/mL measurements obtained through flow virometry, where CC, SC, and DNA exhibited a similar production of 1.06 × 10^10^, 1.05 × 10^10^, and 9.95 × 10^9^ VLPs/mL, respectively.

VLPs were also quantified utilizing nanoparticle-tracking analysis (NTA) (Fig. 1e). ATM, ATR, and PDE conditions presented concentrations of 4.08 × 10^10^, 2.49 × 10^10^, and 2.66 × 10^10^ VLPs/mL in the supernatants at 72 hpt, respectively. These concentrations were significantly higher than the control condition, which exhibited a concentration of 1.21 × 10^10^ VLPs/mL. Conversely, DNA condition did not show a significant difference compared to the control, yielding 1.08 × 10^10^ VLPs/mL in the supernatant. Collectively, three out of the four tested conditions to enhance VLP production were successful, based on the results derived from the three techniques employed to quantify VLPs in the supernatant.

The quality of the VLPs was studied by assessing the particle size distribution in the NTA (Fig. 1e). The mean size (nm) of the produced VLPs for the different knockdowns was: 161 ± 0.95 for DNA (*p*-value = 0.0392), 167 ± 5.8 for ATR (*p*-value = 0.0004), 165 ± 1.2 for PDE (*p*-value = 0.002), and 170 ± 4.4 for ATM (*p*-value < 0.0001). In all cases, knockdown conditions produced particles with slightly higher mean diameters compared to the 153 ± 4.7 nm obtained in the CC and to what was described by González-Domínguez et al. (2020) and by Lavado-García et al. 2020b.

Finally, the relative expression of target proteins was assessed through Western blot analysis. Pixel density analysis of the four target proteins revealed that all shRNA treatments resulted in a 50% or greater knockdown efficacy (Fig. 1f). Specifically, DNA (*p*-value = 0,0062) treatment achieved a relative expression level of 0.5 compared to the control, followed by ATR at 0.38 (*p*-value = 0,0018), ATM at 0.33 (*p*-value = 0,0179), and PDE at 0.23 (*p*-value = 0,0005).

This section evaluated the effects of ATM, ATR, PDEδ, and DNA-PKcs knockdowns on Gag VLP production. Across all conditions, no significant differences in transfection efficiency, viable cell densities, and viabilities were found. VLP production assessment through fluorimetry, flow cytometry, and NTA indicated that ATM, ATR, and PDEδ knockdowns significantly enhanced VLP yields compared to controls, while DNA-PKcs knockdown failed to increase VLP production. Western blot analysis showed a 50% or greater knockdown efficacy for all targeted proteins.

### Assessment of key parameters of Gag VLP production revealed changes in VLP size distribution and secretion upon knockdown

To further investigate the effect of the different knockdowns on VLP production, several key parameters were analyzed: intracellular Gag protein levels, the ratio of Gag protein secreted in the form of VLPs relative to non-secreted Gag, the relationship between specific productivity and particle size distribution, extracellular vesicle (EVs) concentration, and percentage of VLPs relative to the total secreted particles. These measures were assessed in supernatants from 72 hpt cultures.

**Fig. 2:**
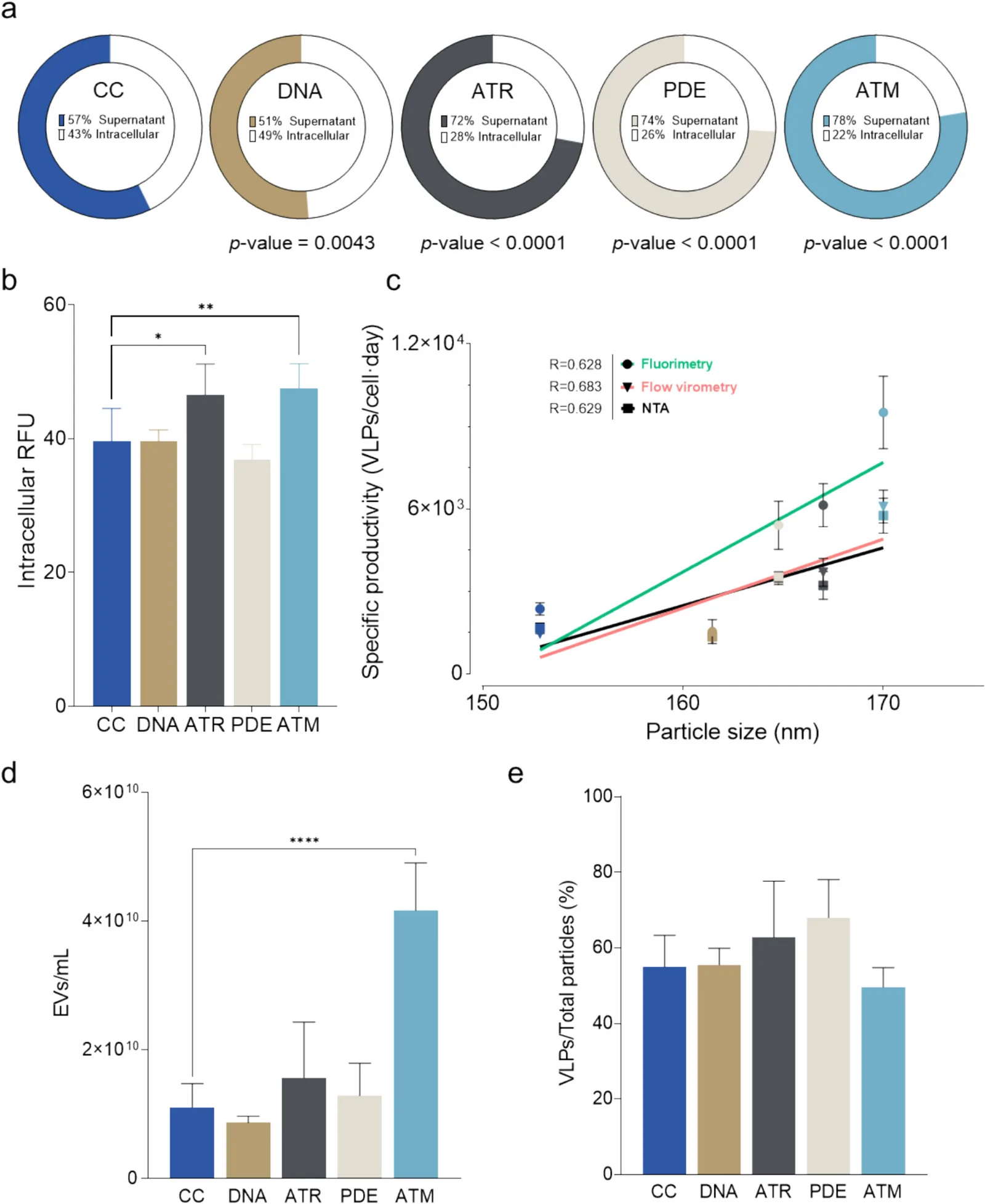
Analysis of key production parameters of HEK293 producing VLPs under transient transfection and target-protein knockdowns. a. Budding efficiency of the different conditions measured as the percentage of RFU in the supernatant relative to the total RFU produced. *p*-values show the statistical significance of the percentage of RFU in the supernatant. b. Intracellular RFU content of the different conditions. c. Regression analysis of the specific productivity (VLPs/cell × day) measured through fluorimetry (dots and green line), flow virometry (inverted triangles and pink line), and NTA (squares and black line) relative to the mean particle size (nm). The points follow the same color legend as in the other panels: navy blue for CC, brown for DNA, grey for ATR, beige for PDE and cyan for ATM. d. Extracellular vesicles (EVs) concentration of the different conditions. e. Percentage of VLPs over the total secreted particles. VLP were measured as fluorescent particles and total particles were measured by dispersion analysis using NTA. In all panels, measurements were carried out using 72 hpt samples. Error bars indicate the standard deviation. Statistical significance was studied using one-way ANOVA and Dunnett test analysis of the knockdowns relative to the control condition (’****’ = *p*-value < 0.0001, ‘**’ = *p*-value = 0.0034, ‘*’ = *p*-value = 0.0118)

In the investigation of the budding efficiency, the ratio of secreted versus non-secreted Gag polyprotein was studied by measuring fluorescence in the cell pellets. Compared to CC, PDE, ATR, and ATM conditions demonstrated a marked increase in the budding efficiency, reflected in a higher ratio of RFU in the supernatant with respect to the intracellular one (Fig. 2a). Conversely, the application of DNA-PKcs knockdown resulted in a decrease in Gag secretion. The proportion of secreted Gag protein was observed to range from 72 to 78% in PDE (*p*-value < 0.0001), ATR (*p*-value < 0.0001), and ATM (*p*-value < 0.0001) conditions. This contrasted with the DNA (*p*-value = 0.0043) condition, which showed only 51% of the RFU present in the supernatant, and the CC, which exhibited 57%. Significantly, only the ATR (*p*-value = 0.0118) and ATM (*p*-value = 0.0034) conditions exhibited an increase in intracellular Gag::eGFP content relative to the CC (Fig. 2b). The intracellular RFU levels in the remaining conditions were maintained at around 40 RFU.

A linear regression analysis was conducted to investigate the relationship between the mean size of the VLPs and their specific productivity, utilizing data derived from fluorimetry, flow virometry, and NTA for each knockdown condition (Fig. 2c). This analysis revealed a strong correlation between the average diameter of the VLPs and the specific productivity, ranging the *R*-squared of the regression models from 0.628 to 0.683. Among the conditions analyzed, ATM exhibited the highest specific productivity and particle size, with values of 7927 VLPs/cell × day and a mean particle diameter of 170 nm. This was followed by ATR, with 4945 VLPs/cell × day and 167 nm; PDE, with 4566 VLPs/cell × day and 165 nm; DNA, with 1433 VLPs/cell × day and 161 nm; and finally, CC, with a specific productivity of 2008 VLPs/cell × day and an average particle diameter of 153 nm. The DNA deviated from the observed linear trend. In this case, lower specific productivity did not lead to a lower particle mean diameter.

NTA was conducted to assess both the concentration of EVs and the percentage of VLPs relative to the total secreted particles (Fig. 2d–e). The EVs are vesicles similar to the VLPs in terms of size and membrane protein composition, making them a difficult-to-purify contaminant in VLPs preparations. The analysis revealed that, with the exception of the ATM condition, knockdowns did not significantly alter the concentration of EVs in the supernatant. Notably, the ATM condition resulted in a significant increase in EV concentration, displaying a 3.8-fold increase (*p*-value < 0.0001) compared to the CC. Regarding the ratio of VLPs to total particles, none of the conditions showed statistical significance. The ATM condition exhibited the lowest percentage of VLP, constituting 50% of the total, followed by CC and DNA conditions at 55%, ATR at 63%, and PDE at 68% (Fig. 2e).

Collectively, an assessment of the effects of various knockdowns on VLP production by analyzing the budding efficiency, EV concentrations, and VLP to total particle proportions in 72 hpt supernatants, was carried out. Notably, PDE, ATR, and ATM conditions significantly increased budding efficiency compared to the control, with DNA knockdown reducing it. ATR and ATM also showed increased intracellular Gag levels. Linear regression revealed a strong correlation between VLP diameter and specific productivity, being ATM the condition that exhibited the highest value. NTA analysis indicated that only the ATM condition significantly increased EV concentration, without significant changes in VLP ratios across conditions. These results highlight the complex effects of genetic knockdowns on VLP production.

### Analysis of the metabolic interactions between knockdowns reveals the pivotal role of ATM

A Box-Behnken experimental design was selected to study metabolic synergies resulting from knockdowns (Cervera et al. 2015a). This response surface method enables to analyze the influence of more than three variables in a given function across three levels while preserving statistical relevance. A plasmid coding a shRNA against histone deacetylase 5 (HDAC5), previously developed and reported by Fuenmayor et al. (2018), was included in the design of experiments (DoE). By doing this, the aim was to augment the targeted bottlenecks and therefore the metabolic synergies for the production of VLPs. The limits of the experimental space were purposely defined to ensure that DNA:PEI ratio was maintained at 1:2, already optimized previously (Cervera et al. 2015a). Consequently, the individual plasmid concentration varied from 0.1 to 0.5 µg/mL, corresponding to a maximal DNA and PEI concentration of 1.6 and 3.2 µg/mL, respectively (Table 1).

**Fig. 3:**
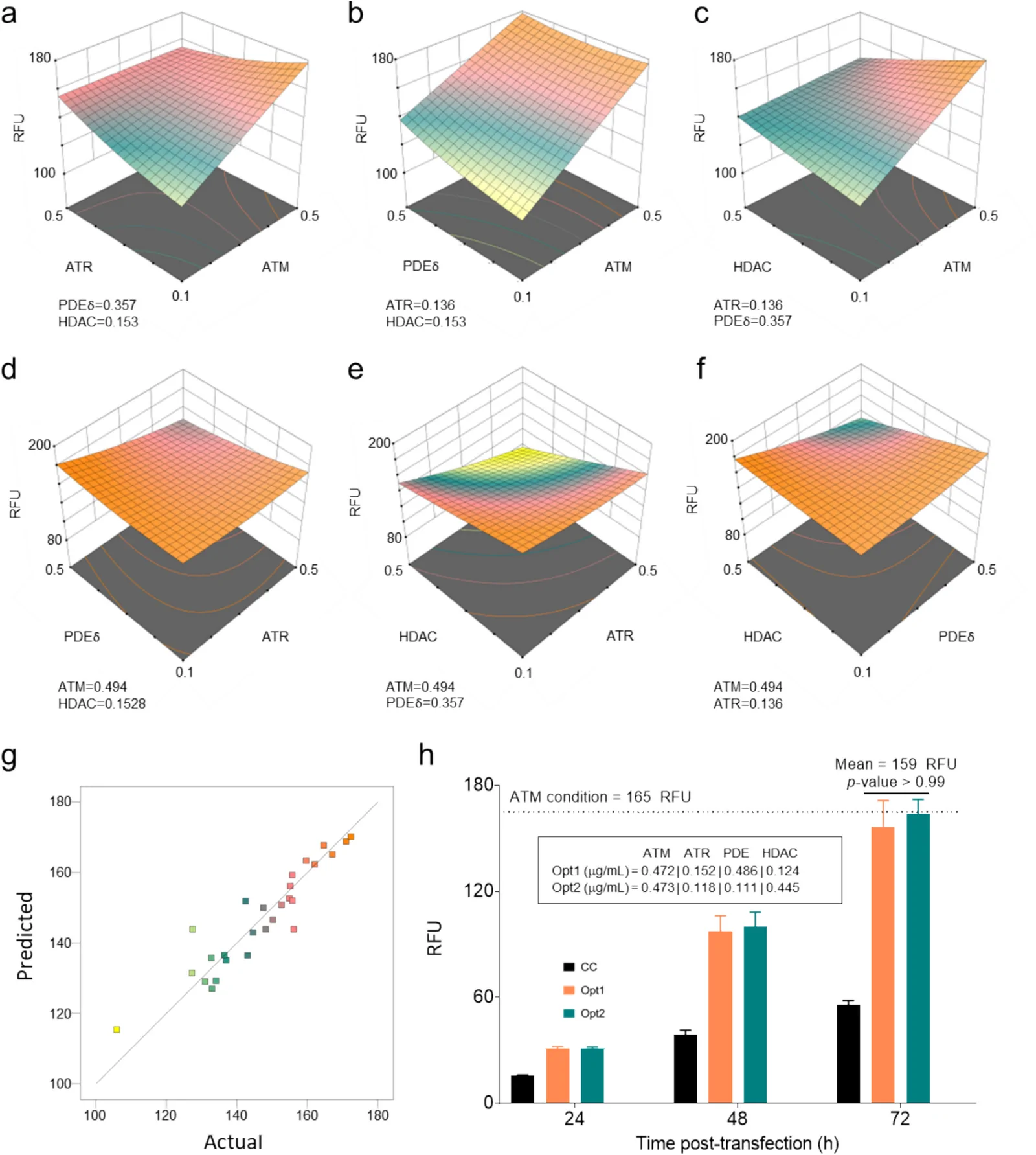
Analysis of the metabolic interactions through Box-Behnken design of experiments. A–f Response surface plots of the response (RFU in 72 hpt supernatants) predicted by the model maintaining two of the four plasmids constant and varying the other two. The concentration of the two plasmids that are kept constant are shown below each plot. g. Plot of the values predicted by the model versus the actual values. h. RFU of the two optimal conditions (Opt) used to validate the model (concentrations of each plasmid are shown inside the box). The dotted line represents the average value of VLP production at 72 hpt using the shATM plasmid alone. Error bars indicate the standard deviation. *p*-value of the statistical analysis (Anova and Dunnett test) comparing RFU in 72 h supernatant of ATM compared to the two optimal conditions is shown in the plot

The analyzed response was RFUs in the supernatant at 72 hpt and the data was fitted to a second-order polynomial equation. The quadratic model (*p*-value < 0.0027), alongside fit statistics and ANOVA analysis, are detailed in Table 1. The analysis of residuals (Fig. 3g) and regression parameters, such as *R*^2^ of 0.8646 and adjusted *R*^2^ of 0.7067 shows that the model is able to accurately predict the experimental data, as can also be observed in the plot of predicted vs actual values (Fig. 3g).

The response surface plots show that in all cases the maximum RFU in the supernatant strongly correlates with ATM and PDE concentrations, observing a broader color shading in the plots with ATM and/or PDE in the x–y axis (Fig. 3a–c) and flatter surfaces when ATM and PDE are fixed at their highest levels (Fig. 3d–f). Additionally, none of the four two-factor interactions studied yielded a positive effect on VLP production, as shown in the model equation, where either non-significant *p*-values or negative terms for the two-factor interaction can be observed.

The model predicted several optimal plasmid ratios, positioning the ATM plasmid near the maximum point of the experimental space (0.5 µg/mL) while adjusting the ratios of other plasmids to achieve a total DNA concentration of approximately 1.2 µg/mL. Two optimal ratios, OPT1 (0.472 µg/mL ATM, 0.152 µg/mL ATR, 0.486 µg/mL PDE and 0.124 µg/mL HDAC) and OPT2 (0.473 µg/mL ATM, 0.118 µg/mL ATR, 0.111 µg/mL PDE and 0.445 µg/mL HDAC), were empirically validated (Fig. 3h), aligning with the prediction of 160 RFU at 72 hpt in both conditions. Still, no significant enhancement in RFUs in the supernatant was observed compared to the ATM knockdown alone, which reached 165 RFU (Fig. 1c–2h) in a regular transfection using 1 µg/mL of total DNA and 2 µg/mL of PEI. Thus, aiming for simplification and cost reduction of the transfection process, subsequent intensification of the metabolic engineering strategy was carried out only using the ATM knockdown.

### Intensification of the Gag VLP production process using a lab-scale stirred tank bioreactor operating in perfusion

Finally, VLP production at bioreactor scale was addressed by combining the ATM knockdown selected as the most promising approach from the performed experiments with the recently developed EGE strategy operating in perfusion mode (Lavado-García et al. 2020a). By combining these two strategies, we aimed to provide the cells with the best production conditions, not only in terms of nutrient availability and transfection efficiency by performing medium exchange and retransfections, but also with optimal gene expression by modulating key pathways for the production of VLPs (Cervera et al. 2015b).

**Fig. 4:**
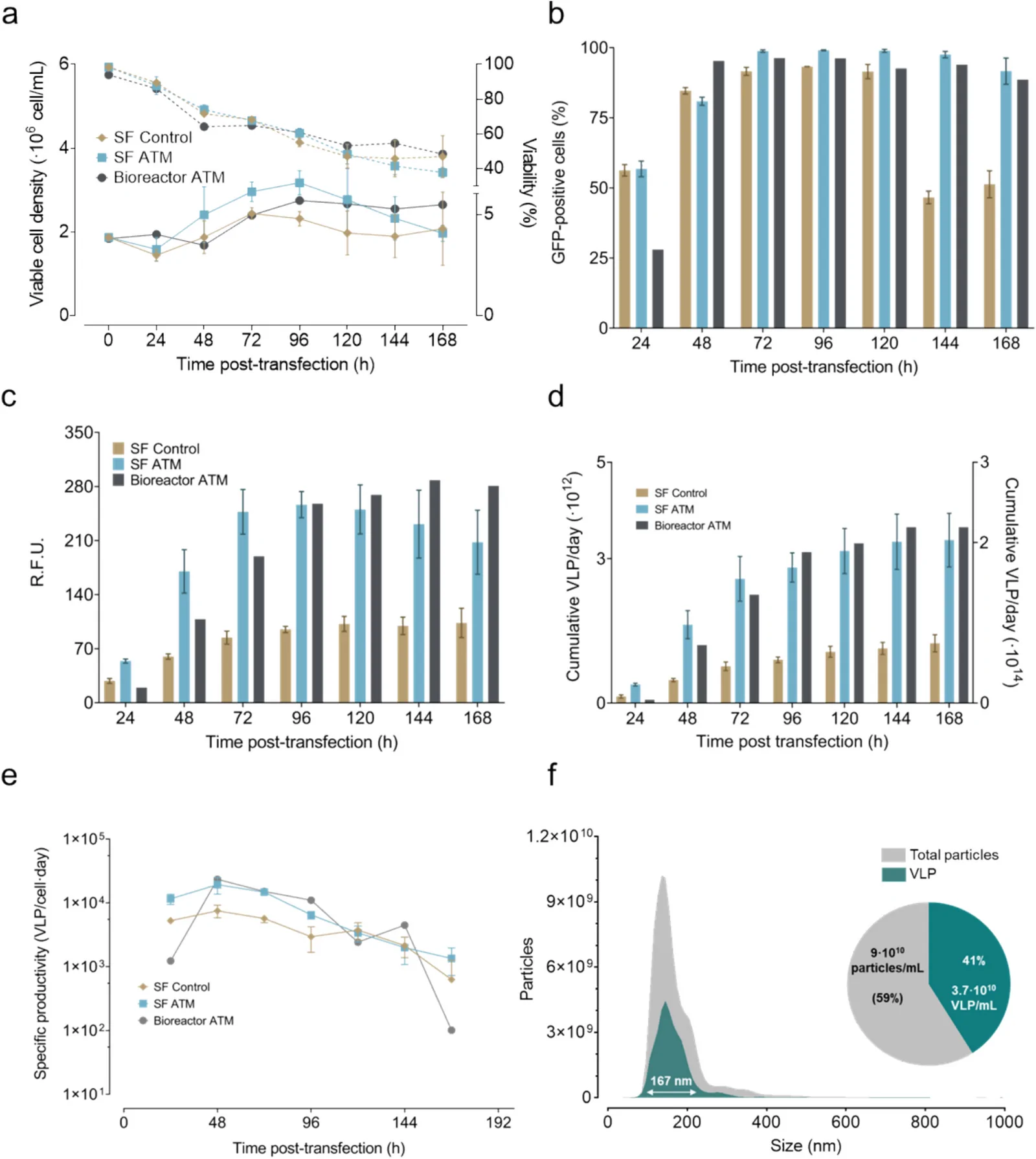
Combination of ATM knockdown with EGE methodology in shake flasks and in bioreactor using perfusion mode. a. Viable cell density (solid lines) and viability (dotted lines) of the different conditions along the studied time course. b. Transfection efficiency measured as GFP-positive cells by flow cytometry every 24 h. c. VLP production of the different studied conditions measured by relative fluorescence units (RFU) in the supernatant along the studied time course. d. Total cumulative VLP concentration calculated as the sum of the VLP in the harvest and the VLP in the supernatants of control condition in shake flask (C_SF_), ATM in shake flask (ATM_SF_) (left y-axis) or in the bioreactor (ATM_B_) (right y-axis). e. VLP specific productivity rate. Experiments in shake flasks performed *n* = 3. Error bars indicate the standard deviation. Experiment in the bioreactor performed *n* = 1. f. Size distribution, average particle diameter, percentage of VLP over total particles and concentration of both fluorescent and total particles in the bioreactor at 168 hpt measured by nanoparticle-tracking analysis

Over a period of 168 h, cell viability and viable cell density (VCD) were monitored for two experimental conditions: ATM protein knockdown in shake flasks (ATM_SF_) and in stirred tank bioreactor (ATM_B_), in comparison to a control condition in shake flasks (C_SF_). Initially, cell viability was approximately 100% across all conditions, followed by a gradual decline to 50% at 168 hpt for ATM_B_ and C_SF_, with a more pronounced decrease to 35% for the ATM_SF_ condition. VCD exhibited a consistent increase until 96 hpt, with the ATM_SF_ condition showing a slightly higher cell density of approximately 3 × 10^6^ cells/mL, compared to both C_SF_ and ATM_B_. Beyond this point, VCD in the ATM_SF_ condition declined, whereas C_SF_ and ATM_B_ conditions reached a plateau at approximately 2 and 2.5 × 10^6^ cells/mL, respectively (Fig. 4a).

At 24 hpt, the ATM_B_ condition showed a transfection efficiency significantly lower than that observed in the C_SF_ and ATM_SF_ conditions (Fig. 4b). This discrepancy disappeared by 48 hpt, after which GFP-positive cells remained above 90% for all conditions until 120 hpt. A notable decrease below 50% was observed in the C_SF_ condition at this point, correlating with a slight increase in its VCD from 120 hpt onwards. In contrast, the ATM_SF_ and ATM_B_ conditions maintained high transfection efficiencies (> 80%) throughout the experiment.

RFUs in the supernatant were measured every 24 h, with peak values of 256 RFU at 96 hpt for ATM_SF_ and 102 RFU at 120 hpt for C_SF_. The ATM_B_ condition achieved a peak of 288 RFU at 144 hpt, indicating a 2.8-fold increase relative to the C_SF_ (Fig. 4c). In all cases, the maximum cumulative VLP (Fig. 4d) was observed at 144 hpt, with no significant increase at 168 hpt.

Daily VLP-specific productivity for the three conditions is presented in Fig. 4e. An initial observation of lower specific productivity between the bioreactor and shake flask conditions correlated with the measurements of RFU and transfection efficiency at 24 hpt. At 48 hpt maximum specific productivities of 19,300 and 23,400 VLPs/cell × day were achieved for ATM_SF_ and ATM_B_, respectively, compared to 7,496 VLPs/cell × day for the C_SF_ condition. This increase in productivity for ATM_SF_ and ATM_B_ relative to C_SF_ was maintained until 120 hpt, after which productivity levels converged and continued to decline until the end of the cultivation. Volumetric productivity in ATM_B_ was 7.5 × 10^12^ VLPs/L × day, significantly higher than the 3.5 × 10^12^ VLPs/L × day obtained in C_SF_ calculated using the RFU.

The quality of the VLPs produced knocking down ATM was analyzed using NTA for the supernatants in the bioreactor at 168 hpt (Fig. 4f). A mean VLP diameter of 155.1 nm was measured, similar to CC (Fig. 1e) and closer to the VLP diameter described in previous works (González-Domínguez et al. 2020; Lavado-García et al. 2020a). The total particle concentration reached 9.0 × 10^11^ particles/mL, with 3.7 × 10^10^ VLPs/mL comprising 41% of the total particles (Fig. 4f).

These results elucidated the impact of the EGE strategy combined with ATM protein knockdown on VLP production, highlighting significant findings in transfection efficiency and VLP-specific productivity in the bioreactor. An overall 3.1-fold increase in cell-specific productivity, a 2.1-fold increase in volumetric productivity, and a 2.8-fold increase in the maximum RFU in the supernatant was achieved following this strategy.

## Discussion

### Effect of knockdown strategies on VLP production in HEK293 cells

Understanding the influence of protein knockdown on VLP production offers insightful observations on cellular mechanisms and their potential exploitation for enhanced bioprocessing. The observations made using the NTA, flow virometry, and fluorometry showed that the downregulation of PDEδ led to a notable 2.2-fold increase in VLP titers in the supernatant. PDEδ’s critical role in modulating the intracellular localization of various proteins, such as solubilizing Rab GTPases implicated in HIV-1 replication, is well-documented (Zaragoza et al. 2005; Caillet et al. 2011; Gerber et al. 2015). Furthermore, recent studies have shown interactions between PDEδ and 2',3'-cyclic-nucleotide 3'-phosphodiesterase (CNPase), highlighting a mechanism through which blocking PDEδ expression could enhance VLP production (Wilson et al. 2012; Ma et al. 2013; Lavado-García et al. 2021a). Like other interferon response proteins such as bone marrow stromal antigen 2 (tetherin), the CNPase interacts directly with the Gag polyprotein in the late phases of the viral cycle, interfering with the budding process. In previous work, it was found that a reduction in the expression of CNPase increased 2.5-fold VLP-specific productivity in HEK293 cells (Lavado-García et al. 2021b). Therefore, PDEδ’s knockdown effect could be attributed to a reduction in CNPase presence at the plasma membrane, potentially alleviating its inhibitory impact on HIV-1 VLP budding. However, elucidating the precise effect of PDEδ on VLP production exceeds the scope of the current study and requires further investigation.

In contrast, the knockdown of DNA-PKcs did not result in an increase in VLP production; rather, a slight decrease in VLP concentration was observed. This result contrasts with previous studies where the inhibition of DNA-PKcs was linked to higher titers of HIV-1 virions (Ellis et al. 2011). This discrepancy may stem from the role of DNA-PKcs in DNA repair following provirus integration (Daniel et al. 2005; Nunnari et al. 2005; Anisenko and Gottikh 2019), a process that is not involved in the VLP production system used in this study, highlighting the need for further research to better understand these dynamics.

An enhanced VLP production was also found under ATR downregulation, with a 2.7-fold increase compared to control conditions. ATR is a phosphatidylinositol 3-kinase-related kinases (PIKK) protein that, together with ATM and DNA-PKcs, is implicated in DNA damage response. ATR contributes to HIV-1 replication, particularly through interactions with viral accessory proteins that were not identified in the production system used here, such as Vpr and Tat (Roshal et al. 2003; Yang et al. 2010; Docando et al. 2020). This suggests a complex relationship with VLP production mechanisms that requires further exploration.

The most substantial increase in VLP titers, a 3.2-fold rise compared to the control, was observed by ATM knockdown. These findings align with previously reported studies indicating ATM's role in metabolic adaptation. Inhibition of ATM has been reported to promote pro-tumorigenic nutrient uptake via mechanisms beyond conventional transporter-mediated pathways. Specifically, it has been described that the inhibition of this protein increases micropinocytosis to promote cell survival through metabolic adaptation to nutrient-poor conditions (Chen et al. 2023). Previous research carried out in our group has shown the importance of micropinocytosis in DNA-PEI complex uptake (González-Domínguez et al. 2019). The upregulation of micropinocytosis, potentially induced by ATM knockdown, may thus serve as a mechanism to enhance the uptake of DNA-PEI complexes as well as nutrient uptake, contributing to both the observed increase in VLP production and the sustained high transfection efficiencies observed in in the VLP production following the EGE protocol, up to 168hpt. This highlights micropinocytosis as a promising target for improving biotechnological applications, particularly in the production of VLPs and potentially other recombinant proteins. Further investigation into the specific pathways influenced by ATM in the context of transfection should be carried out to fully elucidate these mechanisms and their implications in bioprocess development.

### Effect of the knockdowns on key production parameters

Evaluation of several crucial production parameters elucidated notable influences of the knockdowns on the Gag VLP biosynthesis. An enhancement in budding efficiency was observed in the PDE, ATR, and ATM conditions relative to the CC. This enhancement was accompanied by a marked increase in the intracellular levels of Gag polyprotein in the ATR and ATM conditions, alongside a general increase in total fluorescence across all three conditions when compared to CC. Overall, budding efficiency seems to have a positive correlation with the Gag-eGFP production levels, suggesting that the increase in Gag production is likely the primary driver behind the observed alterations in VLP production.

The quantification of the mean particle size by NTA revealed that all experimental conditions resulted in mean diameters between 100 and 200 nm, a size range previously described for HIV-1 VLP (González-Domínguez et al. 2020; Lavado-García et al. 2021b). However, significant differences were observed in the mean particle diameter between the knockdown conditions and the CC. The observed discrepancies in particle size exhibited a positive correlation with specific VLP production rates. In general, higher specific productivities gave rise to higher mean diameters. Particle size has been previously described as a Critical Quality Attribute (CQA) for viral particles and exosomes (van der Pol et al. 2014; Lavado-García et al. 2020a). Variations in the particle size distribution may indicate particle aggregation and changes in the native conformation and in the monomer composition of VLPs (Lavado-García et al. 2021b; González-Domínguez et al. 2021), both of which are crucial parameters that affect the functionality and downstream purification of the particles (González-Domínguez et al. 2021). However, in the absence of detailed insights into the roles of various protein knockdowns within the VLP synthesis pathways, it remains challenging to elucidate the implications of increased mean particle diameter on the overall quality and functional attributes of the VLPs. Further characterization of the VLPs produced under the effect of the different knockdowns should be carried out to assess whether these changes in mean particle size negatively affect VLP integrity and functionality.

An analysis was conducted to examine extracellular vesicle production and the percentage of VLP relative to the total particles generated under the various knockdown conditions. The investigation revealed that none of the tested knockdown conditions significantly altered the ratio of VLPs to total particles produced. However, there was a nominal increase in the percentage of VLP for PDE and ATR knockdowns, while ATM knockdown was associated with a slight decrease. Notably, ATM knockdown resulted in a significant 3.8-fold increase in extracellular vesicle production, an increase of a similar magnitude as the 3.4-fold observed in VLP production. These findings imply that ATM knockdown not only increases Gag polyprotein production and budding efficiency but may also play a role in promoting vesicle secretion.

### Optimization of the metabolic interactions between the different knockdowns

An investigation into the metabolic interactions among ATM, ATR, PDE, and HDAC knockdowns was conducted using a response-surface Box-Behnken design. This approach aimed to explore the combination of plasmid with different knockdowns at different concentrations to identify the optimal gene combination and dosage necessary for an effective metabolic reshaping, targeting various cellular bottlenecks and thereby enhancing VLP production, as demonstrated by Lavado-García et al. (2021a) and Fuenmayor et al. (2018). Contrary to expectations, the model indicated that none of the optimized conditions significantly outperformed the VLP titers achieved with ATM knockdown alone, showing that either negative or non-significant two-factor interactions were taking place. After carefully analyzing the data, several insights emerged to rationalize this observation as discussed below.

Initially, it was hypothesized that the targeted proteins, despite potentially participating in distinct pathways influencing Gag VLP production, might contribute to varying effects on VLP titers. However, this hypothesis remains to be verified. Notably, the knockdown efficiencies of certain proteins, including ATR, HDAC, and PDE, were relatively modest, yet they still led to comparable increases in VLP titers. Consequently, a minor decrease in plasmid concentration might result in an inadequate gene dose for achieving an efficient knockdown, thus impacting VLP production. This scenario is further constrained by the limits on the total amount of DNA in transfections, which is limited due to PEI toxicity, and the necessity to maintain a fixed DNA:PEI ratio of 1:2. As a result, the simultaneous knockdown of several proteins did not yield an additive effect on VLP production, as can be observed in the non-significant *p*-values found in the terms describing the effect of the two-factor interactions in the model equation (Table 1). This could be overcome by including all the shRNAs in the same plasmid, although this strategy would not allow varying the ratios. Another plausible explanation is the presence of other, yet unidentified, metabolic bottlenecks that could cap the VLP production levels, rendering the combined knockdowns less efficient than the ATM knockdown alone.

Given these considerations and the observation that the total DNA required for the optimal conditions was approximately 20% higher than for the ATM knockdown alone, it was decided to focus the metabolic engineering strategy within the EGE protocol exclusively on ATM knockdown. This streamlined approach is based on the understanding that simplifying the genetic intervention may provide a more efficient path to enhancing VLP production without the complexities and potential drawbacks associated with multi-gene knockdown strategies.

### Implementation of the ATM knockdown in the EGE methodology

The implementation of ATM knockdown within the EGE protocol in a stirred tank bioreactor operating under perfusion mode resulted in a notable enhancement of the average specific productivity. This was 2.8 times higher, achieving 8.3 × 10^3^ VLPs/cell × day compared to the previously described 3 × 10^3^ VLPs/cell × day (Lavado-García et al. 2020a). In terms of volumetric productivity, the ATM_B_ knockdown condition yielded 7.5 × 10^12^ VLPs/cell × day, significantly outperforming the 2.7 × 10^12^ VLPs/cell × day described previously (Table 3). Notably, while the ATM condition in shake flask demonstrated lower VCD and cell viability, the bioreactor configuration exhibited superior performance. This improvement is likely attributable to enhanced control over process parameters, such as pH and pO_2_, in addition to the advantages of continuous media exchange compared to the semi-perfusion method used in shake flasks.

**Table 3:** Comparison of key performance parameters of a perfusion-intensified process in the STR using the regular plasmid and the plasmid carrying the shRNA against the ATM protein

Parameter	Lavado et al. (2020)	This work
Accumulated VLPs	8.7·10^13^	2.2·10^14^
Max. VLP concentration in a day (VLPs·mL − 1)	1.5·10^10^	4.4·10^10^
Mean VLP specific productiviy (VLPs·cell − 1·day − 1)	3.0·10^3^	8.3·10^3^
Volumetric productivity/spent medium (VLPs·L − 1·day − 1)	2.7·10^12^	7.5·10^12^
Final (168 hpt) transfection efficiency (%)	45	89
Total volume of spent media (L)	3.8	4.2

In this study, the transfection efficiency observed in ATM knockdown conditions was maintained for a longer duration compared to the control condition. The mechanism underlying the observed increase in GFP-positive cells, however, remains unclear. ATM may be directly or indirectly involved in the transfection process, which may lead to an increase in its efficiency after knockdown. Another hypothesis is that the ATM knockdown may not necessarily enhance transfection efficiency directly but could increase the intracellular accumulation of the Gag::GFP fusion protein as a collateral effect of the important increase in Gag::GFP observed. This accumulation might result in cells that appear GFP-positive in assays, potentially leading to the identification of false positives, cells that are no longer actively transfected but continue to exhibit GFP fluorescence. Further investigation is required to fully understand the impact of ATM knockdown on the cellular processes affecting VLP production under transient transfection.

The final VLP concentration achieved in the bioreactor was 3.7 × 10^10^ VLPs/mL, surpassing the 1.5 × 10^10^ VLPs/mL in the condition without downregulation of ATM. Furthermore, a total of 2.2 × 10^14^ VLPs were harvested at 168 hpt, representing a 2.5-fold increase over the 8.7 × 10^13^ VLPs obtained using the regular EGE protocol in a stirred-tank bioreactor (Table 3). Moreover, the mean particle diameter was 167 nm, concomitant with the observed in the transient gene expression (TGE) protocol (Fig. 1e).

These findings raise important questions about the specificity of the ATM knockdown effect on Gag VLP production pathways. It remains unclear whether the knockdown targets a pathway specific to Gag VLP production or influences a broader range of pathways that could also enhance the production of other recombinant proteins. Clarifying this distinction is crucial for understanding the underlying mechanisms driving the observed increase in productivity. It could also pave the way for developing strategies to leverage ATM knockdown for generating high-yield cell lines. Such approaches would be valuable not only for HIV-1 VLPs but also for a wide range of recombinant proteins and viral vectors. This opens the door for future research to explore the application of ATM knockdown in biotechnological processes beyond the current scope, potentially leading to more productive and affordable recombinant protein production processes.

In this study, specific metabolic engineering approaches of HEK293 cells to enhance VLP production were explored. Three key proteins, that positively influence HIV-1 Gag VLP production were identified: PDEδ, ATR, and ATM. The downregulation of these proteins resulted in respective increases of 2.2-fold, 2.7-fold, and 3.2-fold in VLP titers in the supernatant. Notably, ATM knockdown alone achieved a value of RFUs in the supernatant comparable to those obtained by the combined downregulation of multiple proteins, with a peak of 165 RFU observed at 72 hpt. This suggests that ATM plays a pivotal role in enhancing VLP production.

In addition, the feasibility of ATM downregulation within an intensified cell culture process in a bioreactor has been demonstrated, paving the way for the industrial application of this strategy. The bioreactor process with ATM knockdown maintained transfection efficiencies greater than 80% throughout the duration of the experiment, in contrast to the control condition, which exhibited a significant decline in GFP-positive cells after 120 hpt. The total VLP yield at 168 h was 2.2 × 10^14^, representing a 2.5-fold increase over the yield from previously described strategies.

Overall, our findings indicate that targeted metabolic engineering, especially through ATM knockdown, can significantly improve VLP production efficiency. This includes enhanced product titers, budding efficiency and sustained transfection levels. Such advancements hold promise for developing more efficient vaccine production processes.

## Data Availability

All data used in this study is publicly available.
